# Its International Aims and Position To-Day

**Published:** 1918-10-26

**Authors:** 


### THE HISTORY OF THE RED CROSS SOCIETY.*

### Its International Aims and Position To-Day.

The Red Cross Society is of much more recent date than the Order of St. John. Its origin may be described as an evolution of the feeling of international pity and charity for the sick and wounded soldier, and it is the result of sentiment appealing to science. It was felt that every civilised nation should help to lessen pain and suffering, so as to afford unostentatious yet prompt and systematic relief to the wounded soldier, who may have fallen whilst defending his country's honour; and it was urged that responsible authorities should regard it as a duty to remedy the great evils and horrors arising out of war. The historical records of war from Cyrus to Napoleon have demonstrated the appalling insufficiency of official measures taken to provide relief in war, the wounded being often abandoned inevitably to convents and to charitable persons. The "Memoirs of Larrey" abounded in episodes of fearful suffering. At the siege of Dantzic there were 1,600 wounded and 2,000 sick, yet not a single straw mattress was provided for them to lie upon, no basins to wash their wounds, and no nurses to attend to them. There was no linen, no food, and there were no candles to light the hospitals by night. For five days the wounded never left their wagons, which served as beds as well as transport carts. The carelessness in regard to the sick in the eighteenth and nineteenth, and even in our present century, has been in many instances barbarous, and the improvidence as to medical nursing most culpable.

### Terrors and Neglect of Wounded.

At the taking of Spires in 1792 the wounded were delayed from 24 to 36 hours before removal, and the greater part of the sick and wounded perished. After Waterloo the wounded were conveyed to Antwerp in boats to the places appropriated for them along the Arsenal Quay, but everything for their care and comfort was wanting; there was no lint, linen, bandages, pillows, sheets nor blankets. The complete absence of medical comforts was responsible for the dysentery, cholera, diarrhœa, and other sickness during the Crimean War, and at the Battle of the Alma the wounded men were left exposed for two whole nights on the battlefield. Such neglect was described as the crime of high treason against humanity, yet it will be found on every page of the world's military history. It led a great military medical writer, Dr. Chenu, to state that if the honour and the defence of any State should require of every family, from the most noble to the most humble, the separation from and the sacrifice of a son, a brother, or a father—as is to-day the case in our own country—it must be only on the positive understanding that the State shall take the place of the absent family and shall assure to its defenders prompt and efficacious assistance when this is required. The State must take on those duties which the absent family itself would at any cost fulfil to soothe pain, to save life, or to lessen the agony of any one of its members.

### Need for Special Voluntary Efforts.

In spite of this strong indictment and just demand it is inevitable that during strategical evolutions, and especially after heavy engagements which have cost many lives, more men and more material are required for the sick than any Government can possibly provide; hence the need for special voluntary efforts, and it was the realisation of this imperfect help given to the suffering soldier and the inadequacy of the official means of relief that led M. Henri Dunant, a Swiss, who was a witness of the terrible sufferings endured by the wounded on the battlefield of Solferino in 1859, to make his personal appeal to the Société Génévoise d'utilité publique in 1863 to discuss whether relief societies might not be formed in times of peace so as to render assistance and to provide help to the sufferers, in times of war, by supplying them with the necessary material help served to them by qualified volunteers. As a result of his appeal an International Conference was held in 1863 which issued certain recommendations. These were accepted by the Governments of fourteen different countries at a Congress held in Geneva in 1864, and a Convention was signed and adopted as an "internal code." In consequence, the so-called International Society was formed which established a branch in this country through Lord Wantage in 1870, as the "National Society for Aid to the Sick and Wounded in War." This Society afforded voluntary help for the first time during the Franco-German War, when it supplied 13,000 qualified volunteers between September and December 1870. It has furnished aid in every other war and in every important campaign since then, and it was the parent of the present British Red Cross Society.

### The Birth of the British Red Cross.

In 1898 the Central British Red Cross Council came into being as a Central Council representing the National Aid Society, the St. John Ambulance Association, the Army Nursing Service Reserve, and the St. Andrew's Ambulance Association, but in 1905 under the Presidency of Queen Alexandra these two Societies, viz., the Central British Red Cross Society and the National Society for Aid to the Sick and Wounded in War became fused into one, viz., the British Red Cross Society. As may be known the Red Cross Society is not one independent organisation for relief. It has to receive the seal and the official sanction of the war authorities in each country; neither is it one great international institution embracing all the charitable services of all the different States in one uniform system of philanthropy; each branch in each country has its own national organisation, but with an agreed international object, and, although the end aimed at is the same in all the branches, the means to secure this end may differ. In each country there has been respect for the common aims to be attained, and there has been an acknowledged universal recognition of the neutrality of the Red Cross **until the German barbarians, who claim to be the cultured of the earth, in search of outrage and frightfulness, adopted murder and assassination as means of destruction for neutrals, doctors, nurses, patients, wounded, women, the aged and defenceless, and the children.** Only in this war have hospital ships been sunk at sight with all on board, and only in this war have hospitals been wilfully and deliberately bombed and destroyed. The Huns alone of all the nations have committed with national acclamation and rejoicing such atrocities in war, not forgetting the issue of a medal to celebrate the destruction and murder by U-boats of the s.s. liner *Lusitania*.

* Portion of a paper read by Sir Robert Armstrong Jones, M.D., at the College of Ambulance, Vere Street, W. For other sections of this paper see The Hospital, July 27, 1918, pp. 367 and 368.

